# Changes in Phytochemical, Physiological, and Morphological Traits in *Pelargonium graveolens* as Affected by Drought Stress and *Ascophyllum nodosum* Extract

**DOI:** 10.3390/ijms26189210

**Published:** 2025-09-20

**Authors:** Negar Gerami, Mehdi Rahimmalek, Mahdiyeh Gholami, Behnaz Tohidi, Antoni Szumny

**Affiliations:** 1 Department of Horticulture, College of Agriculture, Isfahan University of Technology, Isfahan 84156-83111, Iran; negar.gerami28974@gmail.com (N.G.); mah.gholami@iut.ac.ir (M.G.); 2 Department of Food Chemistry and Biocatalysis, Wrocław University of Environmental and Life Sciences, 50-375 Wrocław, Poland; 3 Department of Agronomy and Plant Breeding, College of Agriculture, Isfahan University of Technology, Isfahan 84156-83111, Iran; b.tohidi@alumni.iut.ac.ir

**Keywords:** *Pelargonium graveolens*, drought stress, *Ascophyllum nodosum*, physiological, morphological, essential oil

## Abstract

Nowadays, the use of natural biological bio-stimulants such as seaweed extract (SWE) is highly considered for alleviating the adverse effects of drought stress in many plant species. This study evaluated the effects of drought stress and foliar application of seaweed extract (SWE) on the morphological, physiological, and phytochemical traits of *Pelargonium graveolens*. Three levels of water irrigation regimes were used in combination with four SWE concentrations (0, 2.5, 5, and 7.5 mL L^−1^). Based on the GC-MS analysis, 83 compounds were identified, of which citronellol, citronellyl formate, α-gurjunene, δ-cadinene, and γ-cadinene were the major constituents of *P. graveolens* leaves. The highest citronellol content (56.2%) was found under moderate irrigation with 5 mL of L^−1^ SWE, while the lowest amount (26.78%) was obtained under full irrigation with no foliar application of SWE. Citronellyl formate and α-gurjunene exhibited their highest relative abundance under non-stress conditions following foliar application of 5 mL L^−1^ and 0 mL L^−1^ of SWE, respectively. In contrast, δ-cadinene reached its highest value under severe drought stress when treated with 7.5 mL of L^−1^ SWE, indicating a stress-responsive shift in essential oil (EO) composition profile. Principal component analysis (PCA) revealed that full irrigation with 7.5 mL of L^−1^ SWE and mild drought with 5 mL of L^−1^ SWE were the best treatments for ameliorating the EO content and composition. ANOVA revealed that SWE significantly improved the fresh root weight, leaf dimensions, carotenoids, total chlorophyll, protein content, and antioxidant enzyme activities. The 7.5 mL of L^−1^ SWE treatment notably increased fresh root weight by 29.16% and enhanced chlorophyll and protein levels under moderate and severe drought conditions. Drought stress reduced shoot biomass but had no significant effect on chlorophyll content. Carotenoid and antioxidant activities were significantly influenced by both drought and SWE, with the highest levels observed at 5 mL of L^−1^ SWE. Antioxidant enzymes (CAT, SOD, and guaiacol peroxidase) and total antioxidant activity were enhanced by SWE and its interaction with drought stress conditions. These results suggest that foliar SWE application at 5–7.5 mL L^−1^ effectively mitigates drought stress and enhances both growth and EO composition in *P. graveolens*.

## 1. Introduction

*Pelargonium graveolens* L’Hér., a member of the Geraniaceae family, is an aromatic herb native to South Africa, particularly the Comoros Islands, and is now widely cultivated in diverse regions, including Russia, Egypt, Algeria, Morocco, the Congo, Japan, Central America, and several European countries such as Spain, Italy, and France [1]. The plant is characterized by finely divided spiky leaves and small pink flowers. Its essential oil (EO) is highly valued in the perfumery, food, and beverage industries owing to its distinctive fragrance and bioactive compounds [2]. In addition to its commercial uses, EO possesses notable pharmacological properties, including antidepressant, antiseptic, astringent, and hemostatic effects. It has been reported to enhance blood circulation, stimulate adrenal and lymphatic activity, and exhibit diuretic effects, making it beneficial for managing conditions such as fluid retention and cellulite [3].

Water scarcity is a major environmental constraint that poses a serious threat to global agricultural productivity. Drought stress disrupts normal physiological processes in plants, impairs growth, disturbs water relations, and reduces water-use efficiency [4]. This typically occurs when transpiration and evaporation rates exceed the water available in the root zone. Consequently, drought can elicit a wide range of plant responses, including alterations in gene expression, growth inhibition, reduced yield, decreased photosynthetic activity, osmotic adjustment, pigment degradation, and loss of membrane stability [5]. The ability of plants to tolerate drought stress depends on the complex interplay of physiological, biochemical, and molecular mechanisms that regulate growth and development under water-limited conditions [6]. Notably, these physiological responses vary with the severity and duration of drought, underscoring the importance of understanding drought intensity when assessing plant stress responses [7].

Plant secondary metabolites are a diverse class of bioactive compounds including EOs, phenolic acids, flavonoids, alkaloids, and tannins. These natural products are increasingly valued in various industries for their applications in pharmaceuticals, bio-agrochemicals, flavoring agents, and food additives [8,9]. Under drought stress, excessive production of reactive oxygen species (ROS) is commonly observed, leading to oxidative damage to cellular components such as membrane lipids, carbohydrates, proteins, nucleic acids, and DNA [10]. To mitigate ROS-induced damage, plants have evolved to have a complex antioxidant defense system that comprises both enzymatic and non-enzymatic components. Key antioxidant enzymes include superoxide dismutase (SOD), ascorbate peroxidase (APX), catalase (CAT), peroxidase (POX), and glutathione reductase (GR) [11]. In addition to enzymatic defenses, drought stress triggers intracellular signaling cascades that result in altered gene expression and biosynthesis of protective metabolites [12]. Compounds such as glycine betaine, soluble sugars, proline, and abscisic acid are non-enzymatic metabolites that accumulate in response to drought and contribute to osmotic adjustment and stress mitigation [4]. Moreover, drought-induced stress often disrupts membrane structure and stability, which can be manifested by a reduction in photosynthetic pigments, particularly chlorophyll content [13].

In recent years, enhancing drought tolerance through the application of specialized products has emerged as a key agronomic strategy. Various compounds, such as seaweed extracts (SWEs), protein hydrolysates, other nitrogen-containing substances, humic and fulvic acids, chitosan, and other biopolymers, have demonstrated biostimulant properties that promote plant growth and resilience [14]. Among these, the use of bio-elicitors derived from natural extracts, including SWEs, has been proposed as a sustainable approach to improve plant productivity without causing adverse environmental effects [15].

In particular, extracts from the brown seaweed *Ascophyllum nodosum* have been widely studied for their efficacy as biological elicitors in various plant species [15,16,17,18]. These extracts have been shown to enhance plant tolerance to abiotic stresses, including drought [17]. Numerous crops, such as rice [10], tomato [19,20], maize [21], soybean [22,23], onion [24], and coriander [25], have shown that SWE application can improve the drought resistance of crops and reduce the negative impact of drought on crop yield.

The increasing severity of drought conditions in Iran has led to growing interest in *P. graveolens* because of its dual value as both a medicinal and ornamental plant, particularly for use in urban green spaces. Despite extensive research on drought stress mitigation in various crop species, limited attention has been paid to aromatic plants such as *P. graveolens*, particularly in relation to biostimulant-based interventions. This study is the first to investigate the combined effects of drought stress and *A. nodosum* SWE on the physiological, morphological, and phytochemical traits of *P. graveolens*. By focusing on this commercially valuable yet underexplored species, the present study offers novel insights into sustainable strategies for enhancing drought resilience and preserving EO quality under water-limited conditions. To evaluate the effects of SWE and drought on the growth indices and EO composition of *P. graveolens*, an experiment was designed to achieve the following objectives: 1) to investigate the effect of foliar application of SWE on physiological and morphological traits and the enhancement of drought tolerance in *P. graveolens* under varying levels of water stress and 2) to assess the effects of drought and SWE on the EO composition and antioxidant activity of *P. graveolens.*

## 2. Results

### 2.1. Variance Analysis of Morphological Traits

The data from the analysis of variance (ANOVA) are presented in Table 1. ANOVA was performed to assess the effects of drought stress, SWE foliar application, and their interaction on the morphological and physiological traits of *P. graveolens*. The ANOVA results revealed significant differences in fresh root weight (FRW), leaf length (LL), leaf weight (LW), leaf area (LA), total chlorophyll (Chl-t), carotenoids (CAR), protein content (PRO), catalase (CAT), superoxide dismutase (SOD), guaiacol peroxidase (GPx), and antioxidant activity (AA) under different concentrations of SWE application (Table 1). In addition, the effects of drought stress were significant on dry root weight (DRW), fresh shoot weight (FSW), dry shoot weight (DSW), relative water content (RWC), CAR, PRO, and AA, indicating robust activation of defense mechanisms. In addition, the interaction effect between SWE and drought stress was obtained significant for all studied attributes except for FRW, DRW, FSW, PH, LL, LW, LA, and CAR. Plant height was unaffected by any of the treatments (Table 1).

DSW varied markedly across treatments, with the highest values observed under non-stress conditions without SWE foliar application, representing optimal growth in the absence of drought. Under mild drought stress, DSW diminished significantly in the untreated control and at lower SWE concentrations (2.5 and 5 mL L^−1^). In any case, all SWE treatments under mild stress resulted in a restoration of shoot biomass to a level statistically comparable to the non-stressed control, demonstrating a compensatory impact of SWE. Under severe drought stress, DSW was moderately reduced in the control (10.86 g pot^−1^), and only the highest SWE concentration resulted in substantial recovery (15.88 g pot^−1^). The current result shows that using more SWE can reduce the loss of plant weight caused by drought (Table 2). Although LL, LW, and LA reached their highest values with 5 mL L^−1^ SWE application, no further enhancement was observed at the higher concentration of 7.5 mL L^−1^, indicating a possible saturation or inhibitory effect at elevated doses (Table S1). Total chlorophyll content (Chl-t) peaked under normal condition with 2.5 mL L^−1^ SWE (17.05 mg g^−1^ FW), while drought stress reduced chlorophyll levels, especially under severe stress environment with no SWE application (9.70 mg g^−1^ FW), suggesting pigment degradation due to stress. Application of SWE improved chlorophyll retention across all stress levels, with the highest values (16.58 mg g FW^−1^) recorded under mild stress condition with 5 mL L^−1^ SWE. This indicates SWE’s role in preserving photosynthetic capacity under adverse conditions. RWC was negatively affected by drought stress, with the lowest value (65.03%) observed under mild stress (W75) with 5 mL L^−1^ SWE. Under non-stress conditions (W100), RWC remained high across all treatments, with the highest value (74.26%) recorded at 2.5 mL L^−1^ SWE; however, this value was not significantly different from that of the untreated control (73.28%). Under mild and severe stress conditions, SWE application did not result in statistically significant improvements in RWC, although values were generally maintained within a comparable range. These findings suggest that while SWE did not enhance leaf hydration in a statistically measurable way, it may have contributed to the preservation of water status and turgor under drought conditions (Table 2).

The protein content, as a key osmo-protectant, was significantly influenced by SWE, drought, and their interactions. SWE at 5 and 7.5 mL L^−1^ increased the protein content, while 2.5 mL L^−1^ reduced it. The highest protein level (16.66 mg g FW^−1^) was observed under 50% field capacity with 7.5 mL L^−1^ SWE, indicating enhanced osmotic adjustment (Table 2).

CAT activity was markedly influenced by both SWE application and its interaction with drought stress, reaching its peak at 7.5 mL L^−1^ under moderate drought conditions, while the lowest activity was observed under severe stress in the absence of SWE (Table 2). This suggests that SWE enhances the enzymatic antioxidant defense of plants, contributing to the detoxification of reactive oxygen species generated under drought conditions. SOD activity remained relatively stable across treatments, with slight increases under SWE application, peaking at W75 with 5 mL L^−1^ SWE (1.89 U g^−1^ FW min^−1^). The highest values were observed under non-stress conditions with 7.5 mL L^−1^ SWE, indicating a priming effect of SWE on oxidative stress defense mechanisms, even in the absence of drought. GPx activity was the highest (0.07 U g ^−1^ FW min ^−1^) with 2.5 mL L^−1^ of SWE under severe stress. These findings suggest that SWE supports peroxidase-mediated cellular protection, particularly under moderate stress conditions. Finally, AA increased consistently with the SWE concentration across all treatments. The highest value under non-stress condition with 5 mL L^−1^ SWE (97.49%), highlighting SWE’s contribution to cellular resilience under both optimal and stress conditions (Table 2).

### 2.2. Measurement of Secondary Metabolites

Chemical profiling of *P. graveolens* EOs under varying drought intensities and foliar application of SWE revealed significant shifts in the abundance and diversity of volatile compounds (Table 3). These changes reflect both stress-induced metabolic adjustments and modulatory effects of SWE on secondary metabolite biosynthesis. A total of 83 compounds were identified in the EOs, with the most abundant constituents being citronellol (26.78–56.2%), citronellyl formate (8.47–15.61%), α-gurjunene (2.36–6.56%), δ-cadinene (2.40–6.22%), citronellyl tiglate (1.58–6.62%), γ-cadinene (1.20–5.32%), and *cis*-rose oxide (1.40–3.72%). Given the extensive number of detected compounds, this discussion focuses on the key constituents with the highest frequency and relevance.

The percentage of citronellol in the EOs ranged from 26.78% in full irrigation condition and without foliar application of SWE to 56.2% under severe drought (50% field capacity) in treatments with 5 mL L^−1^ SWE followed by 2.5 mL L^−1^ SWE usage (51.2%) (Table 3). Mild drought stress (75% field capacity) increased *cis*-rose oxide content with 5 and 7.5 mL L^−1^ SWE application. Citronellyl formate content ranged from 8.47% to 15.61%, with the highest concentration (15.61%) observed under full irrigation combined with 5 mL L^−1^ SWE. This was followed by 15.07% in the non-stressed condition with 2.5 mL L^−1^ SWE, and 14.93% under mild drought stress with foliar application of 5 mL L^−1^ SWE. The concentration of α-gurjunene was the highest (6.56%) under normal humidity conditions without extract application, whereas it was the lowest (2.36%) under mild stress with the application of 5 mL L^−1^ of SWE (Table 3). α-Gurjunene biosynthesis appears to be downregulated by SWE and drought, possibly due to a shift in metabolic pathways toward more stress-responsive compounds. δ-Cadinene and Citronellyl tiglate also showed their highest amount under severe stress conditions with the application of 7.5 mL L^−1^ of extract.

### 2.3. Principal Component Analysis and Cluster Heat Map of Major Essential Oil Compounds

**Figure 1:**
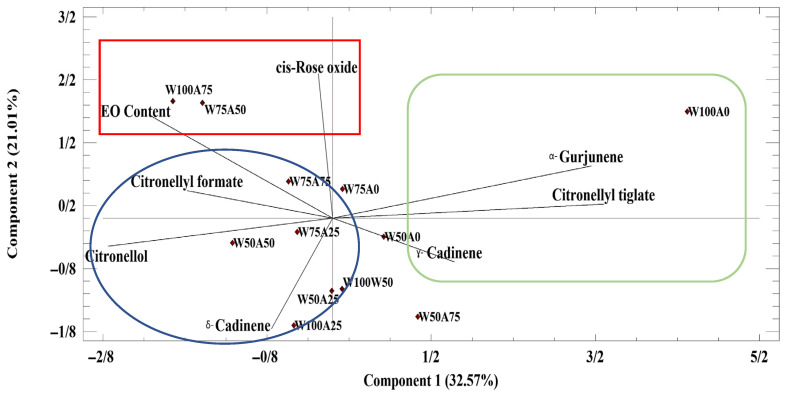
The principal component analysis (PCA) based on major Essential oil components. W50A0: Irrigation at 50% field capacity and foliar application of 0 mL L^−1^ seaweed extract; W75A0: Irrigation at 75% field capacity and foliar application of 0 mL L^−1^ seaweed extract; W100A0: Irrigation at 100% field capacity and foliar application of 0 mL L^−1^ seaweed extract; W50A25: Irrigation at 50% field capacity and foliar application of 2.5 mL L^−1^ seaweed extract; W75A25: Irrigation at 75% field capacity and foliar application of 2.5 mL L^−1^ seaweed extract; W100A25: Irrigation at 100% field capacity and foliar application of 2.5 mL L^−1^ seaweed extract; W50A50: Irrigation at 50% field capacity and foliar application of 5 mL L^−1^ seaweed extract; W75A50: Irrigation at 75% field capacity and foliar application of 5 mL L^−1^ seaweed extract; W100A50: Irrigation at 100% field capacity and foliar application of 5 mL L^−1^ seaweed extract; W50A75: Irrigation at 50% field capacity and foliar application of 7.5 mL L^−1^ seaweed extract; W75A75: Irrigation at 75% field capacity and foliar application of 7.5 mL L^−1^ seaweed extract; W100A75: Irrigation at 100% field capacity and foliar application of 7.5 mL L^−1^ seaweed extract.

Principal component analysis (PCA) was conducted to reduce data complexity and identify treatments that optimize EO content and composition in *P. graveolens*. The biplot, based on the first two principal components (PC_1_ and PC_2_), explained 53.58% of the total variation, indicating a moderate dimensional reduction and meaningful separation of EO composition (Figure 1). The first PC (PC_1_) accounted for 32.57% of the total variation, which was positively correlated with α-gurjunene (0.50), γ-cadinene (0.23), and citronellyl tiglate (0.52). The second component (PC_2_) accounted for 21.01% of the total variance. It also showed a high positive contribution from cis-rose oxide (0/64), α-gurjunene (0.23), and EO content (0.46). Identifying the relationships between the studied variables based on the first two PCs allowed for an obvious separation between these variables.

PCA biplot identified three main groups that were distinguished by their EO composition. Accordingly, *P. graveolens* under W100A0 (100% field capacity + without SWE) and W50A75 (75% field capacity + 7.5 mL L^−1^ SWE) treatment was characterized by a high value of α-gurjunene, citronellyl tiglate, and ɣ-cadinenes. Notably, W50A50 (50% field capacity + 5 mL L^−1^ SWE) was strongly aligned with citronellol, highlighting it as the most effective treatment for maximizing citronellol, a key compound in the economic and aromatic value of EOs. The major samples of the third group were characterized by high levels of EO content and *cis*-rose oxide under mild stress conditions using 5 mL L^−1^ SWE application (Figure 1).

**Figure 2:**
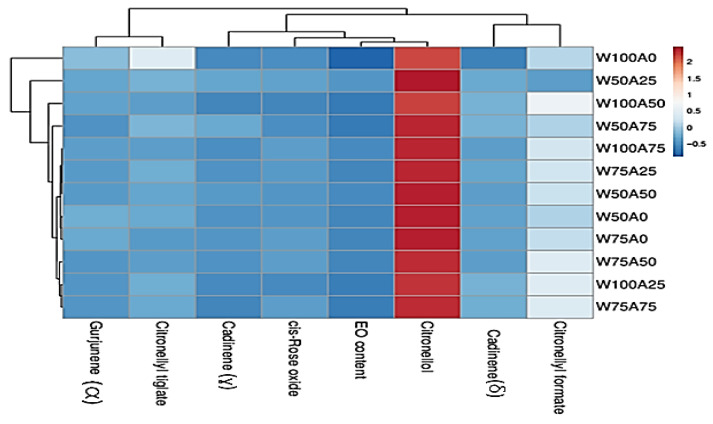
Hierarchical Clustering of essential oil constituents across treatment combinations. W50A0: Irrigation at 50% field capacity and foliar application of 0 mL L^−1^ seaweed extract; W75A0: Irrigation at 75% field capacity and foliar application of 0 mL L^−1^ seaweed extract; W100A0: Irrigation at 100% field capacity and foliar application of 0 mL L^−1^ seaweed extract; W50A25: Irrigation at 50% field capacity and foliar application of 2.5 mL L^−1^ seaweed extract; W75A25: Irrigation at 75% field capacity and foliar application of 2.5 mL L^−1^ seaweed extract; W100A25: Irrigation at 100% field capacity and foliar application of 2.5 mL L^−1^ seaweed extract; W50A50: Irrigation at 50% field capacity and foliar application of 5 mL L^−1^ seaweed extract; W75A50: Irrigation at 75% field capacity and foliar application of 5 mL L^−1^ seaweed extract; W100A50: Irrigation at 100% field capacity and foliar application of 5 mL L^−1^ seaweed extract; W50A75: Irrigation at 50% field capacity and foliar application of 7.5 mL L^−1^ seaweed extract; W75A75: Irrigation at 75% field capacity and foliar application of 7.5 mL L^−1^ seaweed extract; W100A75: Irrigation at 100% field capacity and foliar application of 7.5 mL L^−1^ seaweed extract.

Hierarchical clustering of EO profiles revealed distinct treatment-dependent metabolic patterns (Figure 2). Treatments W100A0, W50A25, and W100A25 formed a closely related cluster characterized by elevated citronellol levels and high EO content, indicating a strong positive association between citronellol accumulation and overall EO content. In contrast, W75A75, W75A25, and W50A50 grouped separately, exhibiting moderate levels of cis-rose oxide, citronellyl tiglate, and cadinenes (γ and δ), suggestive of a more balanced or stress-responsive metabolic profile. W100A75 displayed the highest citronellol and EO content, distinguishing it as a metabolically enhanced condition. Notably, W75A50 and W100W50 clustered independently, driven by elevated citronellyl formate and divergent cadinene levels, implying the activation of alternative biosynthetic pathways. Compound clustering further supported these trends, with citronellol and EO forming a tight cluster, while citronellyl formate, γ-cadinene, and δ-cadinene were grouped together, reflecting shared regulatory mechanisms. These findings underscore the influence of treatment combinations on EO composition, and highlight citronellol as a key determinant of EO productivity.

## 3. Discussion

Roots are the primary organs that sense water deficiency in soil and play a key role in conferring drought stress tolerance to plants [26]. Adequate and accessible moisture in the root zone enhances traits such as the surface area, diameter, weight, and volume of roots [27]. Conversely, drought stress reduces photosynthesis, which can significantly decrease root dry weight [28]. In the present study, DRW increased under severe drought stress (50% field capacity). This reflects a well-established adaptive strategy by which plants enhance water acquisition under limited soil moisture conditions. Drought stress often triggers a shift in biomass allocation, favoring root development over shoot growth to improve access to deeper water reserves and maintain physiological stability [29]. Moreover, increased root tissue density and dry mass have been reported as consistent morphological adaptations to severe drought, contributing to improved mechanical strength and water-uptake efficiency [30]. These findings align with the current results, suggesting that enhanced DRW represents a strategic investment in root architecture to mitigate drought-induced stress and sustain metabolic functions.

In addition, FRW significantly increased in plants sprayed with SWE at a concentration of 7.5 mL L^−1^ compared to that in the control. These findings are consistent with those of previous research on crops such as beans, lentils, and chickpeas, where SWE application significantly promoted root growth and root initiation [31]. Contrary to the present results, Alizadeh et al. [32] reported that 25% and 50% field capacity drought stress significantly reduced the root dry weight in beans. As drought stress intensifies, cell expansion declines, leading to a reduction in fresh root weight [33]. Chen et al. [34] reported that SWE improved soil aggregate structure and promoted root growth to deeper soil to increase water absorption and similar results were observed by Campobenedetto et al. [35] in various tomato genotypes. These findings are likely due to stomatal closure and the reduced activity of photosynthetic enzymes, ultimately decreasing photosynthesis, which is consistent with the present results.

In this study, drought stress in *P. graveolens* reduced RWC, primarily due to lowered leaf water potential. This decline reflects impaired water uptake and increased transpiration loss, which are the typical physiological responses to soil moisture deficits. Drought stress disrupts cellular hydration and turgor maintenance, leading to reduced RWC and compromised photosynthetic efficiency [36]. Similar findings have been reported in transgenic *P. graveolens* lines, where drought stress significantly decreased leaf water potential and RWC, correlating with reduced biomass and essential oil yield [37]. Fan et al. [38] stated that leaf RWC is a useful indicator of plant water status and metabolic activity in plant tissues. According to the present results, RWC values remained statistically indistinguishable between treatments under both mild and severe drought stress, suggesting that SWE did not measurably improve drought-induced RWC reduction. These findings are consistent with previous reports indicating that SWE may help maintain but not necessarily increase. Jacomassi et al. [39] showed that spraying sugarcane plants with SWE helps keep their cells hydrated and boosts their ability to fight stress during dry conditions. This improves their physical strength without greatly changing their water content. Gholamin and Khayatnezhad [40] also noted that the RWC stable during dry conditions is usually linked to the plant’s ability to adjust its internal water levels and protect itself with antioxidants, instead of just taking in more water. Additionally, SWE can maintain turgor pressure, facilitate cell enlargement, and improve plant water relations, thereby enhancing the RWC [41]. Greenhouse studies on vegetables and turf grasses have demonstrated that SWE application enhances water-use efficiency, increases leaf water content, and promotes recovery of wilted plants under drought stress [42]. Based on the present results, under mild stress conditions, a reduction in RWC was observed following SWE application. The reduction in RWC may be attributed to the increased membrane permeability or accelerated metabolic activity during extraction, leading to water loss. Zakaria and Kamal [43] emphasized that SWE modifies the dielectric constant of water, enhancing solubility of both polar and non-polar compounds, but such conditions may also affect tissue hydration dynamics.

Leaf surface area is a key parameter for evaluating plant growth and processes such as photosynthesis [44]. The application of SWE at elevated concentrations has been shown to significantly increase leaf area. This stimulatory effect is attributed to the presence of bioactive compounds, such as cytokinins, auxins, betaines, and micronutrients that promote cell division, elongation, and chloroplast development, all of which influence plant growth through direct or indirect mechanisms [45]. Alizadeh et al. [32] reported that foliar application of brown SWE at concentrations of 0.2%, 0.4%, and 0.6% significantly increased the leaf area in beans. They also observed significant increases in leaf length at 0.2% and 0.4% NaCl concentrations. Similarly, in rose plants, weekly application of 20% SWE significantly increased leaf area, plant biomass, and flower number, whereas both lower and higher concentrations produced suboptimal results owing to insufficient or excessive hormonal input. Thus, foliar application of SWE in *P. graveolens* likely promotes leaf growth by enhancing nutrient uptake and increasing cytokinin content [45].

One of the primary indicators of drought tolerance in plants is the maintenance of photosynthetic pigments under stress [46]. The observed increase in chlorophyll content at the lowest and middle concentrations of SWE under normal and mild stress conditions (Table 2), in contrast to the unchanged levels in the highest concentration treatments, suggests the presence of a threshold beyond which SWE no longer exerts a stimulatory effect. This threshold likely reflects the optimal range of bioactive compounds—such as cytokinins, betaines, and micronutrients—that enhance chlorophyll biosynthesis by promoting nutrient uptake, enzymatic activity, and chloroplast development [47,48]. At moderate doses, these compounds act synergistically to improve photosynthetic efficiency; however, when SWE is applied at higher concentrations, the physiological balance may be disrupted. Excessive SWE can lead to osmotic stress, hormonal oversaturation, or nutrient antagonism, all of which may impair pigment synthesis or stability [49,50]. Similar dose-dependent responses have been reported in various crops, where biostimulant efficacy diminishes or reverses at supra-optimal levels [51,52]. Elevated chlorophyll levels observed under severe stress conditions in response to increased SWE application suggest a protective role of the pigment, likely contributing to improved stress tolerance. In contrast, under mild stress, the physiological damage may not have reached a threshold that triggers strong compensatory pigment synthesis, and lower SWE concentrations may have been sufficient to elicit a significant response. Thus, the enhancement of chlorophyll content under severe stress appears to be both dose-dependent and stress-intensified, reflecting the synergistic effect of SWE bioactive and plant stress signaling pathways.

The current findings are consistent with those of previous studies that reported significant reductions in chlorophyll and carotenoid content under severe drought stress. Similarly, in rice (*Oryza sativa*), foliar application of SWE led to a dose-dependent increase in chlorophyll and carotenoid levels, suggesting improved pigment biosynthesis and protection against oxidative stress [53]. These effects are attributed to SWE’s ability of SWE to modulate hormonal signaling (e.g., cytokinins and abscisic acid), upregulate stress-responsive genes, and improve water retention and nutrient uptake [47]. The synergistic action of the SWE components supports pigment stability and chloroplast integrity, thereby counteracting drought-induced pigment loss and restoring photosynthetic function. Furthermore, SWE has cytokinin-like activity, which may delay chlorophyll degradation and increase chloroplast division [54]. Kumari et al. [55] reported that seaweed extracts improved photosynthetic efficiency by enhancing the chlorophyll composition. In 2013, Latique et al. [56] suggested that increased chlorophyll levels in seaweed-treated bean plants might be due to the presence of glycine betaine, which reduces chlorophyll degradation. Rouphael et al. [28] also found that seaweed-treated plants had higher chlorophyll content and better photosynthetic performance than controls.

In this study, a reduction in soil moisture to 50% field capacity significantly decreased the protein content of *P. graveolens*. However, bioactive compounds in SWE can enhance nutrient absorption, stimulate the biosynthesis of enzymes essential for protein production, and promote plant growth [57]. Other studies on potato and artichoke have also reported increased amino acid content following SWE application [58,59]. In agreement with these results, obtained findings showed that increasing the concentration of seaweed extract led to higher protein levels in *P. graveolens*. Drought stress negatively affects plant growth, productivity, and survival through the induction of oxidative stress. Plants activate antioxidant mechanisms to mitigate reactive oxygen species (ROS). DaCosta and Huang [60] reported reduced activities of superoxide dismutase (SOD) and catalase (CAT) under prolonged drought stress. Ping et al. [61] also found that catalase activity did not increase during drought. Tatari et al. [62] noted that extended drought reduced catalase activity in three turf grass species. The variation in catalase response may be attributed to differences in plant species, sampling time, or drought severity [63]. In the current study, catalase activity did not change significantly, which is consistent with the findings of Ping et al. [61].

SOD is a key component of the plant defense system and acts as the first line of defense against ROS [64]. In the present study, foliar application of SWE increased both SOD and CAT activities in *P. graveolens*, consistent with the results reported by Sabah [65].

Amiri et al. [66] reported that EO content in *P. graveolens* increased significantly under 75% field capacity compared to well-watered conditions. The observed variation in citronellyl formate content from 8.47% to 15.61%reflects a strong interaction between water availability and SWE application. The highest concentration observed under full irrigation with 5 mL L^−1^ SWE suggests that optimal hydration combined with bioactive supplementation enhances monoterpene ester biosynthesis. This aligns with the finding that SWE can stimulate secondary metabolite production by modulating hormonal signaling and improving nutrient uptake under favorable conditions [67]. Previous studies have reported similar enhancements in terpenoid profiles in SWE-treated *Mentha* and *Ocimum* species, which were attributed to improved photosynthetic efficiency and enzyme activation involved in terpene biosynthesis [68]. Drought stress typically downregulates secondary metabolism owing to reduced carbon assimilation and altered redox balance [67]. However, SWE appears to buffer these effects, possibly through osmoprotective compounds and antioxidant enzyme stimulation. Chai et al. [68] reported that moderate water stress can maintain or even enhance certain quality traits when combined with bio-stimulants. Moreover, citrus rootstocks under drought stress have demonstrated genotype-specific resilience, maintaining metabolic activity and volatile production when supported by exogenous treatments [69]. The observed downregulation of α-gurjunene biosynthesis under drought stress and SWE application suggests reprogramming of secondary metabolism, likely favoring the production of compounds more directly involved in stress mitigation. Such metabolic shifts are consistent with drought-induced modulation of terpenoid pathways, where the biosynthetic flux is redirected toward volatiles with antioxidant properties [70]. In contrast, δ-cadinene and citronellyl tiglate reached their highest concentrations under severe drought conditions combined with 7.5 mL L^−1^ SWE, indicating that SWE may potentiate the accumulation of specific stress-responsive volatiles. δ-cadinene has been associated with enhanced defense signaling and antimicrobial activity, whereas citronellyl tiglate is known for its role in plant–insect interactions and potential antioxidant effects [71]. Eiasu et al. [72] found the highest EO content under alternate irrigation schedules, with increased citronellol and citronellyl formate under stress, and decreased geraniol and geranyl formate. The effect of irrigation level on oil yield and composition was attributed to drought-induced changes in enzymatic activity and metabolism. Consistent with these findings, the current study showed that under normal and mild stress conditions, SWE at high concentrations increased the EO percentage (Table 3). The differential accumulation of these compounds highlights the nuanced role of SWE in modulating drought-induced metabolic responses and supports its potential as a biostimulant for enhancing stress resilience.

## 4. Materials and Methods

### 4.1. Plant Material and Culture Conditions

The study was conducted from 2021 to 2022 in an experimental greenhouse at Isfahan University of Technology, Isfahan, Iran. *P. graveolens* plants were initially obtained in pots measuring 40 cm in height and 30 cm in diameter. Rooted seedlings were transplanted into pots filled with a soil mixture composed of soil, sand, and manure at a 3:1.5:2 ratio (*v*/*v*). To ensure proper establishment, the plants were irrigated regularly for four months. The SWE used in this study was a liquid formulation derived from *A. nodosum*, produced by Acadian Seaplants (Canada). The extract contained 3% potassium and 10% organic matter. The experiment employed a factorial arrangement in a randomized complete design (RCD) with three replications to investigate the effects of drought stress and *A. nodosum* SWE treatments.

#### Treatments and Stress Conditions

The plants were subjected to three irrigation regimes: full irrigation (control) and two levels of deficit irrigation representing moderate and severe drought stress, in combination with four concentrations of SWE application (0, 2.5, 5, and 7.5 mL L^−1^). The drought treatments began in July 2021. Irrigation scheduling was based on soil water depletion: 100% (control), 75% (moderate stress), and 50% (severe stress) of the total available water from the root zone (60 cm depth). The soil moisture content was monitored using the standard gravimetric method [73] at three soil depths: 0–20 cm, 20–40 cm, and 40–60 cm. Irrigation depth for each treatment was calculated using the following formula:

I = (θFc − θirri) × D × (pb/pw)(1)

In this equation, I represents the irrigation depth (cm), θFc is the gravimetric soil moisture content at field capacity, θirri is the gravimetric soil moisture content at the time of irrigation, D denotes the root zone depth (cm), pb is the soil bulk density (1.4 g cm^−3^), and pw is the density of water. Irrigation water was supplied through a pumping system using polyethylene pipes and the volume of water applied to each treatment was measured using a volumetric flow meter.

The plants were treated with Stimplex, a commercial SWE derived from A. nodosum (produced in Canada and supplied by the Arman Sabz Adineh Company, Tehran, Iran). The physicochemical characteristics of the soil in the greenhouse experiment are listed in Table 4. For each application, the SWE solution was freshly prepared prior to the foliar spraying. Treatments were applied every five days, totaling ten applications. After a period of three months, leaf samples were collected and immediately stored at −80 °C for subsequent biochemical and physiological analyses.

### 4.2. Measurement of Morphological and Physiological Parameters

Morphological and physiological characteristics of *P. graveolens* were evaluated under three different moisture regimes and varying levels of SWE application. The assessed morphological traits included leaf length (LL), leaf width (LW), leaf area (LA), fresh (FRW) and dry root weights (DRW), as well as fresh and dry shoot weights (FSW and DSW). FSW and DSW were determined by weighing immediately after harvest and after oven drying at 70 °C to a constant weight, respectively.

#### 4.2.1. Chlorophyll Content Estimation

Chlorophyll (Chl-t) extraction was performed according to the method described by Lichtenthaler [74]. Fresh leaf samples (0.2 g) were ground in a mortar with 20 mL 80% acetone until complete decolorization of the tissue was achieved. The resulting homogenate was centrifuged at 2000× *g* for 10 min. The absorbance of the supernatant was measured at 663 nm, 646 nm, and 470 nm using a UV-600A spectrophotometer. The concentrations of chlorophyll a, chlorophyll b, total chlorophyll, and carotenoids (CAR) were calculated based on the empirical formulas provided by Lichtenthaler [74]:

Chl− a = 11.24 × (A661.6) − 2.04 × (A644.8)(2)

Chl− b = 20.13 × (A644.8) − 4.19 × (A661.6)(3)

Chl− t = 7.05 × (A 661.6) + 18.09 × (A 644.8)(4)

Car (mg g^−1^ FW) = 1000A470 − 1.82 Chl.a − 85.02 Chl.b/198 (5)

#### 4.2.2. Relative Water Content

Relative water content (RWC) was used to assess leaf water status following the method described by Cherki et al. [75]. Fresh leaf samples were collected from each treatment and their fresh weight (FW) was measured immediately. The samples were then immersed in distilled water for 24 h to reach full turgidity, after which the turgid weight (TW) was recorded. Subsequently, the samples were oven-dried at 70 °C for 24 h to determine the dry weight (DW). RWC was then calculated using the following formula:

RWC (%) = [(Fw − Dw)/(Tw − Dw)] × 100(6)

#### 4.2.3. Protein Content

The protein concentration in the plant extracts was determined using the Bradford method, and a colorimetric assay was performed using a spectrophotometer [76]. A 50 µL aliquot of the plant extract was transferred into a test tube, followed by the addition of 3 mL of Bradford reagent. The mixture was vortexed thoroughly and allowed to react for 10 min at room temperature. Absorbance was measured at 595 nm, and the protein content (mg/mL) was calculated using a standard calibration curve.

#### 4.2.4. Antioxidant Enzymes Assay

Fresh leaf tissue (0.2 g) from each treatment was homogenized using a mortar and pestle. Subsequently, 2 mL of sodium phosphate buffer (10 mM, pH 7.0), containing 50 mM potassium phosphate, 1 mM EDTA, 2% polyvinylpyrrolidone (PVP), 2 mM α-dithiothreitol (DTT), 50 mM Tris-HCl, and 0.2% Triton X-100, was added to the homogenate. The mixture was then centrifuged at 12,000× *g* for 30 min, and the resulting supernatant was collected for antioxidant enzyme assays.

Catalase (CAT) activity was determined according to the method described by Agarwal et al. [77]. Briefly, 2 mL of the extraction buffer was combined with 3.8 μL of 30% H_2_O_2_, and 10 μL of the enzyme extract was added to initiate the reaction. The decrease in absorbance was monitored at 240 nm every 30 s for 2 min, using a spectrophotometer.

Superoxide dismutase (SOD) activity was measured using a modified method of Giannopolitis and Ries [78]. For this assay, 50 μL enzyme extract was added to 3 mL reaction buffer containing 50 mM phosphate buffer (pH 7.8), 75 nM EDTA, 13 mM methionine, 63 μM nitro blue tetrazolium (NBT), and 1.3 μM riboflavin. The reaction mixtures were then exposed to white light (50 W) for 15 min. Following illumination, absorbance was measured at 560 nm using a spectrophotometer. A sample that was not exposed to light served as a blank, and a control sample containing all reaction buffer components except the enzyme extract was used as the reference. SOD inhibits photochemical reduction in NBT by preventing the oxidation of riboflavin. During the absorbance measurements, the blank exhibited the least color change, whereas the control showed the most intense color development. Finally, enzyme activity was expressed in enzyme units and calculated based on the following formula:

(7) $$\mathrm{SOD}\;\mathrm{activity}\;=\;\;\frac{100-[(OD\;control-OD\;sample)/Od\;control]\times 100}{50}$$

Guaiacol peroxidase (GPx) activity was measured following the method of Chance and Maehly [79]. For this purpose, 3 mL of reaction buffer-comprising 100 mM sodium phosphate buffer, 15 mM hydrogen peroxide, 3.9 µL guaiacol, and 0.05 mL of enzyme extract was mixed. Enzymatic activity was determined by monitoring the increase in absorbance at 470 nm over a period of two minutes using the following equation:

GPX activity (U) = (Δ_A_ × T_V_ × D)/(ε × V) (8)

where Δ_A_ is the change in absorbance at 470 nm per minute, T_V_ is the total volume of the reaction buffer and enzyme extract (3 mL), V is the volume of enzyme extract used (50 µL), ε is the extinction coefficient of guaiacol peroxidase, and D is Dilution factor.

#### 4.2.5. 2,2-Diphenyl-1-Picrylhydrazyl (DPPH) Assay

The DPPH free radical scavenging activity of the plant extracts was assessed according to the method described by Kloareg Quatrano [80]. To prepare the DPPH solution (0.1 mM), 0.0098 g DPPH powder was dissolved in 80% methanol and brought to a final volume of 250 mL in a volumetric flask. For the assay, 100 µL of plant extract (0.1 mL) was added to 10 mL of DPPH solution in a test tube and incubated in the dark for 30 min. After incubation, absorbance was measured at 517 nm using a spectrophotometer. Antioxidant activity was expressed as the percentage of DPPH radical inhibition, calculated using the following equation:

(9) $$\%\mathrm{inhibition}\;=\;\frac{A_{B}-A_{A}}{A_{B}}\times 100$$

where A_A_ and A_B_ are the absorbance values of the DPPH radical in the presence of the plant extract sample and control, respectively.

### 4.3. GC-Ms Analysis

The *P. graveolens* (scented geranium) leaves were shade-dried at room temperature (20–25 °C) over a period of seven days. The dried leaves were placed in a 500 mL distilltion flask containing 300 mL of distilled water to hydro-distillation for 4 h using a Clevenger-type apparatus. Since the EO is less dense than water, it accumulated on the water’s surface. After distillation, the EO was collected from the apparatus and transferred to dark glass vials for storage at 4 °C. To ensure complete recovery of EO residues from the distillation column, 2 mL of diethyl ether was used for rinsing. Because EO is soluble in diethyl ether, the collected mixture was placed under a fume hood for 12 h to allow the solvent to evaporate, leaving behind the pure EO. EO content was determined using the European Pharmacopeia (ver. 8.2, monograph 2.8.12) on the basis of dry matter. EO content (%) was measured using the following formula [81]:

(10) $$\%\;\mathrm{EO}\;\mathrm{content}\;=\;\frac{Mass\;of\;EO\;obtained\;(g)}{Mass\;of\;dry\;matter\;(g)}\times 100$$

The chemical composition of the EO was analyzed using gas chromatography–mass spectrometry (GC-MS) with the Shimadzu GC-MS QP 2020 PLUS system (Kyoto, Japan), performed in Poland. Compound separation was performed using a Zebron ZB-5 MSi capillary column (30 m × 0.25 mm × 0.25 µm; Phenomenex, Torrance, CA, USA). GC-MS analysis was conducted under the following conditions: the scan range was set from *m*/*z* 35 to 320, with a scan rate of 3 scans per second. Helium served as the carrier gas at a constant flow rate of 1.01 mL·min^−1^, and the injection was carried out in the split mode at a ratio of 1:30. The oven temperature program began at 45 °C, ramped to 150 °C at 2 °C min^−1^, followed by a rapid increase to 270 °C at 15 °C·min^−1^, which was maintained for 5 min. A sample volume of 1 µL was then injected. The mass spectrometer operated with an ion source temperature of 240 °C and an electron ionization voltage of 70 eV. Retention indices of the volatile compounds were calculated based on the retention times of a homologous series of n-alkanes (C7–C24), following the method described by Adams [82] and cross-referenced with the NIST spectral database, as detailed in the HS-SPME Arrow analysis section. Quantification of EO components was achieved by integrating GC-MS peak areas using Spectrus software (version B.05.00).

### 4.4. Statistical Analysis

Data obtained from *P. graveolens* were analyzed based on factorial experiments in a completely randomized design using SAS version 9.4. (SAS Institute, Cary, NC, USA) with three replications. Means were compared using the least significant difference (LSD) test at *p* ≤ 0.05 level of probability. Principal component analysis (PCA) was performed to determine the associations between variables using Statgraphics software version 18.2.04. Cluster analysis was conducted using R software (version 4.2.1) to evaluate the clarity in the EO content and major compositions under water deficit conditions by using different SWE concentrations.

## 5. Conclusions

This study highlights the potential of SWE as a natural and effective tool for improving the growth and stress tolerance of *P. graveolens*. The application of SWE helped the plants cope better with drought conditions by enhancing their physiological functions and antioxidant defense systems. The current findings show that foliar application of SWE, particularly at concentrations of 5 and 7.5, significantly improved various physiological, morphological, and biochemical traits of *P. graveolens* under different levels of drought stress. SWE enhanced root fresh weight, leaf dimensions, leaf area, total chlorophyll, carotenoid, protein content, and antioxidant enzyme activities (CAT, SOD, and GPx), indicating a marked improvement in plant resilience to drought-induced oxidative stress. In addition, SWE improved the quality and quantity of EOs, which are important for the plant’s commercial and medicinal value. These findings suggest that seaweed extract is a promising and eco-friendly approach for supporting the cultivation of *P. graveolens* in areas facing water scarcity.

## Figures and Tables

**Table 1:** Variance analysis of physiological and morphological traits in *Pelargonium graveolens* under water stress and SWE treatments.

Source of Variation	Mean Squares								
	DF ^1^	FRW ^3^	DRW ^4^	FSW ^5^	DSW ^6^	RWC ^7^	PH ^8^	LL ^9^	LW ^10^
SWE ^2^	3	22.17 *	0.83 ^ns^	25.30 ^ns^	7.31 ^ns^	9.22 ^ns^	75.49 ^ns^	1166.21 **	169.81 **
Drought	2	22.17 ^ns^	3.30 *	118.27 ^**^	43.90 **	58.47 **	41.87 ^ns^	22.32 ^ns^	2.13 ^ns^
Drought × SWE	6	5.83 ^ns^	0.61 ^ns^	27.94 ^ns^	12.56 *	33.76 *	35.86 ^ns^	49.85 ^ns^	35.46 ^ns^
Error	24	7.35	0.85	17.96	3.81	10.03	36.52	134.18	16.21
CV (%)	-	12.57	15.99	9.57	14.46	4.53	13.28	9.76	6.10
**Source of Variation**	**Mean Squares**								
	**DF ^1^**	**LA ^11^**	**Chl-t ^12^**	**CAR ^13^**	**PRO ^14^**	**CAT ^15^**	**SOD ^16^**	**GPx ^17^**	**AA ^18^**
SWE **^2^**	3	921,334.60 **	29.54 **	5.74 **	97.4 **	665.65 **	0.27 **	0.0008 **	27.74 **
Drought	2	34,287.13 ^ns^	6.15 ^ns^	1.57 *	10.70 *	35.45 ^ns^	0.009 ^ns^	0.00004 ^ns^	28.28 **
Drought × SWE	6	205,586.22 ^ns^	8.53 **	0.49 ^ns^	9.12 *	154.24 **	0.01 **	0.001 **	3.94 **
Error	24	97,154.21	1.99	0.34	2.9	26.84	0.004	0.00002	0.92
CV (%)	-	12.79	9.82	10.74	14.09	17.81	3.71	14.39	1.01

^1^ DF: Degree of Freedom; ^2^ SWE: Seaweed Extract; ^3^ FRW: Fresh Root Weight; ^4^ DRW: Dry Root Weight; ^5^ FSW: Fresh Shoot Weight; ^6^ DSW: Dry Shoot Weight; ^7^ RWC: Relative Water Content; ^8^ PH: Plant Height; ^9^ LL: Leaf Length; ^10^ LW: Leaf Width; ^11^ LA: Leaf Area; ^12^ Chl-t: Total Chlorophyll Content; ^13^ CAR: Carotenoids; ^14^ PRO: Protein; ^15^ CAT: Catalase; ^16^ SOD: Superoxide Dismutase; ^17^ GPx: Guaiacol Peroxidase; ^18^ AA: Antioxidant activity. ^ns^, non-significant; *, significant at *p* ≤ 0.05; **, significant at *p* ≤ 0.01.

**Table 2:** Mean comparisons of drought stress and SWE (interaction) on morphological and physiological traits of *Pelargonium graveolens*.

Drought	SWE Concentration ^1^	DSW ^2^ (g pot^−1^)	Chl-t ^3^ (mg g^−1^ FW)	RWC ^4^ (%)	PRO ^5^ (mg g^−1^ FW)	CAT ^6^ (U g ^−1^ FW min ^−1^)	SOD ^7^ (U g ^−1^ FW min ^−1^)	GPx ^8^ (U g ^−1^ FW min ^−1^)	AA ^9^ (%)
W100 ^10^	A0 ^13^	16.34 ^a^	13.50 ^cde^	73.28 ^ab^	11.21 ^cde^	27.50 ^def^	1.47 ^e^	0.05 ^b^	95.83 ^b^
	A25 ^14^	14.99 ^a^	17.05 ^a^	74.26 ^a^	8.46 ^ef^	24.06 ^efg^	1.84 ^bc^	0.03 ^cd^	96.60 ^ab^
	A50 ^15^	14.86 ^a^	15.79 ^abc^	73.88 ^a^	16.47 ^a^	33.81 ^bcd^	1.87 ^ab^	0.03 ^cd^	97.49 ^a^
	A75 ^16^	13.74 ^a^	12.89 ^de^	68.16 ^bcd^	14.60 ^ab^	30.94 ^cde^	1.98 ^a^	0.01 ^e^	96.80 ^ab^
W75 ^11^	A0	10.51 ^bc^	12.04 ^ef^	73.37 ^ab^	11.89 ^bcd^	21.77 ^fgh^	1.49 ^e^	0.05 ^b^	92.63 ^cd^
	A25	9.41 ^c^	15.76 ^abc^	68.38 ^bcd^	8.48 ^ef^	18.33 ^gh^	1.85 ^b^	0.03 ^cd^	93.87 ^c^
	A50	10.03 ^bc^	16.58 ^ab^	65.03 ^d^	13.94 ^abc^	36.67 ^bc^	1.89 ^abc^	0.01 ^e^	96.45 ^ab^
	A75	14.4 ^a^	14.86 ^abcd^	67.07 ^cd^	15.96 ^a^	46.41 ^a^	1.73 ^d^	0.03 ^c^	96.41 ^ab^
W50 ^12^	A0	10.86 ^bc^	9.70 ^f^	66.17 ^cd^	8.40 ^ef^	13.75 ^h^	1.53 ^e^	0.01 ^e^	90.63 ^e^
	A25	14.86 ^a^	13.93 ^cde^	67.53 ^cd^	8.13 ^f^	24.64 ^efg^	1.82 ^bc^	0.07 ^a^	91.91 ^de^
	A50	15.07 ^a^	14.23 ^bcde^	70.81 ^abc^	10.78 ^def^	41.83 ^ab^	1.88 ^ab^	0.02 ^d^	95.60 ^b^
	A75	15.88 ^a^	16.38 ^ab^	70.25 ^abcd^	16.66 ^a^	29.22 ^cdef^	1.74 ^cd^	0.03 ^cd^	96.37 ^ab^

In each column, means followed by the same letter are not significantly different according to LSD test at 0.05. ^1^ SWE: Seaweed Extract, ^2^ DSW: Dry Shoot Weight, ^3^ Chl-t: Total Chlorophyll Content, ^4^ RWC: Relative Water Content, ^5^ PRO: Protein, ^6^ CAT: Catalase, ^7^ SOD: Superoxide Dismutase, ^8^ GPx: Guaiacol Peroxidase, ^9^ AA: Antioxidant Activity. ^10^ W100: Irrigation at 100% field capacity; ^11^ W75: Irrigation at 75% field capacity; ^12^ W50: Irrigation at 50% field capacity; ^13^ A0: No foliar apllication; ^14^ A25: Foliar application of 2.5 mL L^−1^ seaweed extract; ^15^ A50: Foliar application of 5 mL L^−1^ seaweed extract; ^16^ A75: Foliar application of 7.5 mL L^−1^ seaweed extract.

**Table 3:** Volatile component (%) of essential oils of *Pelargonium graveolens* under drought conditions and *Ascophyllum nodosum* treatments.

	Treatment	RI^exp^/RI^lit 1^	W_50_A_0_^2^	W_75_A_0_^3^	W_100_A_0_^4^	W_50_A_25_^5^	W_75_A_25_^6^	W_100_A_25_^7^	W_50_A_50_^8^	W_75_A_50_^9^	W_100_A_50_^10^	W_50_A_75_^11^	W_75_A_75_^12^	W_100_A_75_^13^
Components (%)														
**EO Content (%)**		**-**	**0.05**	**0.15**	**0.13**	**0.15**	**0.16**	**0.15**	**0.24**	**0.57**	**0.20**	**0.23**	**0.25**	**0.74**
Acetic acid, pentyl ester		910/911	0.04	0.04	0.04	0.05	0.03	0.01	0.02	0.02	0	0.02	0.01	0.02
3-Heptanone, 5-methyl-		942/944	0	0	0.01	0	0	0.01	0	0	0	0.01	0.01	0.01
2-Heptanol, 6-methyl-		955/958	0.01	0.01	0.02	0.01	0.01	0.01	0.01	0.01	0.01	0.01	0.02	0.01
2-Butenoic acid, 2-methyl-,		960/962	0.09	0.09	0.09	0.11	0.09	0.04	0.06	0.05	0.02	0.08	0.07	0.04
1-methylethyl ester														
5-Hepten-2-one, 6-methyl-		984/986	0	0	0.01	0	0	0.01	0	0.01	0.01	0	0.01	0.01
Myrcene		990/991	0.02	0.16	0.02	0.13	0.22	0.59	0.28	0.04	0.69	0.43	0.53	0.08
Decane		1000/1000	0	0	0.14	0.01	0.01	0.01	0.01	0.04	0.02	0.03	0.01	0.02
δ-3-Carene		1010/1011	0	0	0.02	0	0	0	0	0	0.02	0	0.02	0
p-Cymene		1022/1024	0	0	0.01	0.01	0.01	0.01	0.01	0.01	0.04	0.01	0.05	0.01
Eucalyptol		1024/1025	0.19	0.03	0.03	0.05	0.03	0.03	0.05	0	0.05	0.04	0.04	0.03
β-cis-Ocimene		1035/1038	0.02	0.06	0.01	0.06	0.08	0.26	0.01	0.02	0.41	0.25	0.34	0.04
Benzeneacetaldehyde		1042/1045	0	0	0.04	0.02	0.01	0.01	0.01	0.06	0.01	0	0.02	0.04
β-trans-Ocimene		1047/1049	0.05	0.09	0.01	0.07	0.18	0.42	0.20	0.02	0.57	0.23	0.34	0.04
5-Heptenal, 2,6-dimethyl-		1050/1053	0	0	0.01	0.02	0.02	0.02	0.01	0.02	0.02	0.01	0.03	0.01
γ-Terpinene		1057/1060	0	0	0.01	0	0.01	0.02	0.01	0.02	0.01	0.01	0	0.01
*cis*-Linalool oxide (furanoid)		1072/1075	0.02	0.03	0.02	0.01	0.01	0.01	0.02	0.04	0.04	0.01	0.02	0.02
1-Octanol		1068/1070	0	0	0.01	0	0	0	0.01	0.01	0.02	0.01	0	0
*trans*-Linalool oxide (furanoid)		1082/1086	0.03	0.04	0.03	0.04	0.05	0.06	0.05	0.05	0.16	0.04	0.09	0.03
Linalool		1098/1099	0.30	0.45	0.29	0.31	0.33	0.42	0.40	0.29	1.10	0.31	0.41	0.30
***cis*-Rose oxide**		**1108/1110**	**1.98 ±** **0.05**	**2.86 ±** **0.05**	**3.04 ±** **0.02**	**1.94 ±** **0.04**	**2.36 ±** **0.05**	**1.40 ±** **2.12 ±**	**2.12 ±** **0.06**	**3.72 ±** **0.02**	**1.39 ±** **0.07**	**2.28 ±** **0.02**	**3.61 ±** **0.10**	**2.93 ±** **0.10**
*trans*-Rose oxide		1125/1127	0.95	1.43	1.50	1.02	1.63	0.74	1.21	1.87	0.84	1.22	1.95	1.54
Camphor		1141/1144	0	0.09	0.51	0.05	0.09	0.06	0.05	0.01	0.12	0.06	0.30	0.11
*trans* Verbenol		1142/1145	0.01	0.01	0.02	0.01	0.01	0.03	0.01	0.01	0.03	0.01	0.02	0.02
Citronellal		1151/1154	0.32	0.47	0.27	0.46	0.46	0.06	0.49	0.55	0.33	0.39	0.62	0.66
*cis*-dihydro-β-Terpineol		1158/1160	0.06	0.10	0.19	0.13	0.16	0.21	0.23	0.31	0.34	0.07	0.17	0.15
*iso*-Menthone		1159/1162	1.65	1.87	2.60	1.93	2.02	2.15	2.80	4.50	1.35	1.12	2.20	3.44
*neoiso*-Isopulegol		1170/1172	0.32	0.31	0.07	0.04	0.04	0.02	0.04	0.04	0.03	0.03	0.02	0.04
*iso*-Menthol		1180/1182	0.04	0.05	0.08	0.04	0.05	0.07	0.05	0.05	0.07	0.07	0.11	0.06
α-Terpineol		1186/1190	0.30	0.47	0.10	0.05	0.04	0.01	0.01	0.05	0.14	0	0.13	0.04
Dodecane		1200/1200	0.25	0.42	0.23	0.24	0.12	0.01	0.15	0.13	0.45	0.46	0.44	0.11
*trans*-Piperitol		1205/1207	0.05	0.08	0.08	0.06	0.06	0.07	0.07	0.09	0.05	0	0.03	0.06
*trans* Oct-2-enyl acetate		1207/1209	0.04	0.04	0.04	0.04	0.05	0.04	0.05	0.07	0.08	0.03	0.06	0.04
**Citronellol**		**1225/1228**	**41.33 ±** **0.80**	**40.85 ±** **2.76**	**26.78 ±** **0.83**	**51.20 ±** **2.84**	**42.50 ±** **0.27**	**40.42 ±** **2.30**	**56.20 ±** **0.90**	**41.28 ±** **1.88**	**34.03 ±** **0.01**	**37.59 ±** **0.02**	**38.31 ±** **0.26**	**47.24 ±** **1.49**
Geranial		1265/1270	0.08	0.08	0.04	0.11	0.09	0.06	0.12	0.13	0.03	0.06	0.05	0.16
**Citronellyl formate**		**1273/1275**	**9.01 ±** **1.01**	**10.45 ±** **0.26**	**8.47 ±** **1.01**	**10.44 ±** **0.80**	**12.05 ±** **1.00**	**15.07 ±** **2.76**	**14.89 ±** **0.14**	**14.93 ±** **0.21**	**15.61 ±** **1.42**	**9.51 ±** **0.32**	**13.74 ±** **0.19**	**13.70 ±** **2.30**
Bornyl acetate		1283/1284	0.01	0.06	0.18	0	0.02	0.60	0.04	0.07	0.02	0.03	0.07	0.01
Thymol		1290/1292	0.12	0.56	0.05	0.08	0.10	0.10	0.65	0.80	1.26	0.18	0.21	0.02
p-Menth-1-en-9-ol		1294/1296	0.53	0.62	0.46	0.83	0.88	0.89	0.99	0.97	1.51	1.94	0.85	0.53
Geranyl formate		1298/1299	0.20	0.20	0.19	0.30	0.30	0.30	0.20	0.30	0.45	0.10	0.30	0.22
*trans*-dihydro-α-Terpinyl acetate		1302/1302	0.32	0.40	0.65	0.12	0.28	0.32	0.18	0.15	0.73	0.20	0.32	0.16
α-Terpinyl acetate		1348/1353	0.78	0.95	0.79	0.85	1.25	1.50	0.99	1.02	1.65	1.16	1.36	0.90
Citronellyl acetate		1352/1356	0.30	0.30	0.70	0.40	0.40	0.42	0.56	0.56	0.42	0.56	0.35	0.45
α-Copaene		1374/1376	0.20	0.27	0.20	0.19	0.19	0.15	0.16	0.32	0.15	0.34	0.15	0.27
Geranyl acetate		1382/1383	0.01	0.04	0.03	0.03	0.03	0.04	0.03	0.05	0.03	0.03	0.01	0.04
**Gurjunene <alpha->**		**1407/1409**	**4.31 ±** **0.06**	**4.21 ±** **0.86**	**6.56 ±** **0.13**	**2.47 ±** **0.12**	**2.49 ±** **0.22**	**2.75 ±** **0.13**	**2.91 ±** **0.14**	**2.36 ±** **0.10**	**4.31 ±** **0.19**	**2.91 ±** **0.10**	**2.91 ±** **0.12**	**3.21 ±** **0.12**
*E*-β-Caryophyllene		1417/1419	0.04	0.04	0.13	0.02	0.02	0.02	0.02	0.03	0.03	0.01	0.01	0.03
β-Copaene		1432/1433	1.10	1.05	1.01	1.57	1.65	2.10	1.56	1.62	2.36	2.05	2.07	1.44
Citronellyl propionate		1444/1445	0	0.10	0.03	0.12	0.01	0.01	0.01	0.17	0	0.01	0.03	0
*trans*-Muurola-3,5-diene		1451/1452	0.43	0.59	4.93	0.69	0.68	1.13	0.98	0.84	1.31	0.99	1.14	0.77
α-Humulene		1453/1455	0.10	0.11	0.18	0.10	0.10	0.11	0.14	0.14	0.04	0.11	0.11	0.13
9-epi-(E)-Caryophyllene		1464/1465	0.01	0.01	0.15	0.01	0.15	0.13	0.01	0	0.02	0.01	0.02	0
*cis*-Muurola-4(14),5-diene		1466/1467	0.39	0.39	0.52	0.39	0.41	0.44	0.31	0.44	0.75	0.44	0.31	0.41
Dauca-5,8-diene		1472/1473	0.20	0.20	0.48	0.02	0.03	0.02	0.02	0.03	0.02	0.04	0.01	0.02
γ-Muurolene		1477/1480	1.48	1.76	1.58	1.96	1.98	1.80	2.02	2.44	1.94	1.80	1.44	2.38
Germacrene D		1480/1482	0.04	0.04	0.39	0.09	0.06	0.08	0.03	0.12	0.08	0.06	0	0.09
α-Amorphene		1483/1485	0.04	0.26	0.27	0.26	0.29	0.29	0.34	0.23	0.46	0.49	0.41	0.22
γ-Amorphene		1494/1495	0.34	0.46	0.63	0.42	0.46	0.43	0.60	0.64	0.60	0.60	0.70	0.71
Viridiflorene		1496/1498	0.09	0.11	0.09	0.16	0.18	0.20	0.16	0.15	0.16	0.24	0.27	0.13
α-Muurolene		1501/1500	0.25	0.13	2.58	0.19	0.25	0.18	0.31	0.25	0.34	0.32	0.29	0.18
**γ-Cadinene**		**1513/1514**	**1.20 ±** **0.20**	**1.29 ±** **0.60**	**5.32 ±** **0.10**	**1.42 ±** **0.01**	**2.14 ±** **0.32**	**2.46 ±** **0.25**	**1.42 ±** **0.02**	**1.47 ±** **0.38**	**2.43 ±** **0.45**	**2.76 ±** **0.60**	**2.19 ±** **0.20**	**1.20 ±** **0.10**
**δ-Cadinene**		**1522/1524**	**3.26 ±** **0.10**	**3.34 ±** **0.20**	**2.40 ±** **0.84**	**3.58 ±** **0.30**	**3.78 ±** **0.56**	**6.03 ±** **0.45**	**3.58 ±** **0.21**	**3.68 ±** **0.38**	**5.80 ±** **0.73**	**6.22 ±** **0.83**	**5.58 ±** **0.60**	**3.39 ±** **0.51**
*trans*-Cadina-1,4-diene		1532/1533	0.03	0.03	0.06	0.05	0.05	0.06	0.06	0.06	0.08	0.09	0.12	0.05
α-Cadinene		1537/1339	0.21	1.22	0.15	0.22	0.30	0.37	0.30	0.21	2.01	0.31	0.30	0.16
α-Calacorene		1544/1545	0.13	0.23	0.13	0.26	0.26	0.30	0.28	0.29	0.31	0.40	0.23	0.23
*epi*-Longipinanol		1564/1563	0.74	0.77	0.50	1.22	1.25	1.22	1.05	1.14	1.18	1.59	1.03	1.15
Geranyl butanoate		1566/1567	0.11	0.12	0.08	0.15	0.23	0.22	0.18	0.12	0.20	0.25	0.18	0.11
(2*E*)-Tridec-en-1-ol		1570/1571	1.02	1.29	0.58	1.32	1.54	1.97	1.31	1.01	1.88	2.52	1.48	1.12
Spathulenol		1575/1675	1.12	1.29	2.97	0.83	1.26	1.75	1.05	1.09	1.23	2.18	1.36	1.17
Caryophyllene oxide		1584/1583	0.15	0.16	0.39	0.22	0.25	0.22	0.21	0.21	0.03	0.29	0.20	0.17
Viridiflorol		1591/1593	0.46	0.50	0.52	0.53	0.62	0.65	0.54	0.50	0.27	0.73	0.61	0.49
Humulene epoxide II		1607/1608	0.30	0.30	0.30	0.46	0.49	0.52	0.40	0.32	0.39	0.60	0.40	0.30
1-*epi*-Cubenol		1617/1619	0.45	0.58	0.31	0.75	0.95	0.99	0.75	0.60	0.65	1.05	0.89	0.52
Citronellyl pentanoate		1625/1625	0.65	0.51	0.53	0.67	0.65	0.62	0.73	0.50	0.75	1.09	0.61	0.41
Epicubenol		1628/1629	0	0	0.02	0.02	0	0.03	0.02	0.03	0.01	0.08	0	0.02
τ-Muurolol		1640/1641	0.04	0.05	0.06	0.07	0.05	0.04	0.05	0.05	0.04	0.17	0.04	0.04
Himachalol		1652/1654	0.05	0.05	0.27	0.07	0.08	0.10	0.06	0.05	0.05	0.09	0.08	0.04
Selin-11-en-4-α-ol		1657/1657	1.76	1.28	2.18	0.43	0.15	0.12	0.23	0.38	0.02	0.21	0.05	0.34
**Citronellyl tiglate**		**1665/1668**	**4.02 ±** **0.82**	**3.35 ±** **0.72**	**1.58 ±** **0.62**	**4.62 ±** **0.56**	**4.85 ±** **0.31**	**5.32 ±** **0.22**	**4.52 ±** **0.31**	**3.35 ±** **0.44**	**3.90 ±** **0.21**	**6.62 ±** **0.33**	**4.88 ±** **0.24**	**3.28 ±** **0.32**
Bulnesol		1669/1672	0	0	0.55	0.02	0.02	0.02	0.01	0.01	0	0.01	0.02	0.01
Shyobunol		1687/1689	0.85	0.97	0.42	1.07	1.10	1.32	1.45	0.84	0.94	1.82	1.32	0.74
Geranyl tiglate		1695/1696	0.75	0.86	0.23	1.42	1.25	0.96	0.93	1.10	0.81	1.24	0.99	1.12

^1^ RI exp.–retention indices, experimental according to series n-alkanes; RIlit.: literature retention indices (NIST23 and Adam’s Library); ^2^ W50A0: Irrigation at 50% field capacity and foliar application of 0 mL L^−1^ seaweed extract; ^3^ W75A0: Irrigation at 75% field capacity and foliar application of 0 mL L^−1^ seaweed extract; ^4^ W100A0: Irrigation at 100% field capacity and foliar application of 0 mL L^−1^ seaweed extract; ^5^ W50A25: Irrigation at 50% field capacity and foliar application of 2.5 mL L^−1^ seaweed extract; ^6^ W75A25: Irrigation at 75% field capacity and foliar application of 2.5 mL L^−1^ seaweed extract; ^7^ W100A25: Irrigation at 100% field capacity and foliar application of 2.5 mL L^−1^ seaweed extract; ^8^ W50A50: Irrigation at 50% field capacity and foliar application of 5 mL L^−1^ seaweed extract; ^9^ W75A50: Irrigation at 75% field capacity and foliar application of 5 mL L^−1^ seaweed extract; ^10^ W100A50: Irrigation at 100% field capacity and foliar application of 5 mL L^−1^ seaweed extract; ^11^ W50A75: Irrigation at 50% field capacity and foliar application of 7.5 mL L^−1^ seaweed extract; ^12^ W75A75: Irrigation at 75% field capacity and foliar application of 7.5 mL L^−1^ seaweed extract; ^13^ W100A75: Irrigation at 100% field capacity and foliar application of 7.5 mL L^−1^ seaweed extract. The main Essential oil components are bold and used in analysis.

**Table 4:** Chemico-physical characteristic of soil in the greenhouse experiment.

Soil Texture	Soil-Sand-Manure
Volumetric moisture at field capacity (%)	28.2
Volumetric moisture at the wilting point (%)	14.6
Soil acidity	7.8
Electrical conductivity of soil saturated extract (ECe)	2.55
Organic materials (%)	1.5
Lime	34.5
Nitrogen (%)	0.247
Absorbable phosphorus (mg/kg soil)	49.5
Absorbable potassium (mg/kg soil)	166
Iron (mg/kg soil)	23.4
Zinc (mg/kg soil)	20

## Data Availability

The data will be available based on request.

## Supplementary Materials

The following supporting information can be downloaded at: https://www.mdpi.com/article/10.3390/ijms26189210/s1.
