# Supplementary material for: Changes in Phytochemical, Physiological, and Morphological Traits in Pelargonium graveolens as Affected by Drought Stress and Ascophyllum nodosum Extract
Source: Int J Mol Sci. 2025 Sep 20;26(18):9210. doi: 10.3390/ijms26189210 (PMC12470972; doi:10.3390/ijms26189210)
Supplement: Supplementary file 1 [file ijms-26-09210-s001.zip › ijms-3825938-supplementary.pdf]

Table S1.

Mean comparisons of drought stress and seaweed extract (SWE) on morphological and physiological traits of *Pelargonium graveolens.*

SWE (mL L^{-1})	FRW[3]	LL[5]	LW[6]	LA[7]	[8]CAT
0	19.461 ^{b}	107.45^{b}	60.55 ^{b}	2033.20^{c}	4.3 ^{b}
2.5	21.68^{ab}	110.60^{b}	64.26^{b}	2333.5^{bc}	5.67^{a}
5	21.9^{ab}	131.20^{a}	70.09^{a}	2750.6^{a}	6.11^{a}
7.5	23.25 ^{a}	125.06^{a}	68.67^{a}	2624.1^{ab}	5.78a
Drought	FSW[1]	DRW[2]	CAR[4]		
Severe stress	60.87^{b}	6.12^{a}	5.07 ^{b}		
Mild stress	64.80^{a}	6.07^{a}	5.79^{a}		
Non-stress	67.07^{a}	5.19^{b}	5.42^{a}		

In each treatment means followed by a same letter are not significantly different according to LSD's test at 0.05.

[1]**FRW**: Fresh Root Weight; [2]**DRW**: Dry Root Weight; [3]**FSW**: Fresh Shoot Weight; [4]**CAR:** caretonids; [5]**LL**: Leaf Length; [6]**LW**: Leaf Width; [7]**LA**: Leaf Area; [8]**CAT**: Catalase.
